# Love over gold and mind over matter? Identifying capabilities that preserve medical assistants’ sustainable employability

**DOI:** 10.1186/s12960-024-00937-6

**Published:** 2024-07-22

**Authors:** Bram P. I. Fleuren, Alden Yuanhong Lai, Lynda Gruenewald-Schmitz, Jennifer Larkin, Christina T. Yuan

**Affiliations:** 1 Department of Work and Social Psychology, Faculty of Psychology and Neuroscience, Maastricht University, Maastricht, The Netherlands; 2 Department of Public Health Policy and Management, School of Global Public Health, and Department of Management and Organizations, Stern School of Business, New York University, New York, NY USA; 3 Embrace Leadership Consulting, Hales Corners, WI USA; 4 NCH Healthcare System, Naples, FL USA; 5 Department of Health Policy and Management, Johns Hopkins Bloomberg School of Public Health, Baltimore, MD USA

**Keywords:** Medical assistants, Burnout, Intention to quit, Job satisfaction, Meaningful work, Sustainable employability

## Abstract

**Background:**

Medical assistants (MAs) are crucial for affordable, high-quality primary care, but what motivates this low-wage occupational group to stay in their job remains underexplored. This paper identifies the work aspects that MAs value (“capabilities”), and how they affect sustainable employability, which refers to employees’ long-term ability to function and remain in their job.

**Methods:**

We used structural equation modelling to assess how capabilities relate to four outcomes among MAs: burnout, job satisfaction, intention to quit, and experiencing work as meaningful.

**Results:**

We find that earning a good income, developing knowledge and skills, and having meaningful relationships at work relate to the outcomes. Meaningful relationships represent a stronger predictor than salary for one’s intention to quit.

**Conclusions:**

Competitive salaries are necessary but not sufficient to motivate low-wage health care workers like MAs to stay in their job. Health care leaders and managers should also structure work so that MAs can foster meaningful relationships with others as well as develop competencies.

## Introduction

Medical assistants (MAs) are allied health personnel who provide administrative and clinical support, most notably in primary care clinics in the United States [1]. With relatively low labor costs, MAs form an effective solution in delivering high-quality care in low-margin healthcare segments where staffing resources are limited [2]. They also contribute to team communication and quality of care [3]. As labor demand for MAs continues to increase [4], understanding the factors behind the recruitment and retention of this increasingly prominent health care occupation is important especially as they relate to cost-effective staffing models in ambulatory settings like primary care.

Despite their importance, MAs remain an understudied group of primary care workers, with only a few studies investigating their occupational functioning and well-being. A few qualitative studies reported that high workload, low pay, and lack of recognition [5] are relevant to MAs’ occupational functioning and well-being, as are relationships with colleagues and patients, job control, and self-efficacy [6]. One quantitative study reported organizational culture and low self-efficacy as central to MA burnout [7]. Besides these studies, research on MAs remains largely a-theoretical, qualitative, or restricted to one domain of occupational functioning (e.g., burnout). The result is a long list of factors that either promote or hinder MA’s occupational functioning and well-being, with limited insights into the interrelationships among factors or underlying mechanisms. Aiming to advance theorizing on the occupational dynamics of low-wage healthcare workers, Lai and colleagues recently proposed using the capability approach to study MAs [8]. This approach highlights seven capabilities that people value universally at work (e.g., the capability to use knowledge and skills), which when achieved, can promote employees’ long-term ability to function in their jobs [9, 10]. This paper extends Lai and colleagues’ work by testing the capability approach in the context of an occupation that has been characterized as low-wage or low-skill in nature [8, 11]. Specifically, using structural equation modeling, the paper investigates which of the seven capabilities are most relevant to MAs’ job outcomes like burnout, job satisfaction, intention to quit, and work meaningfulness.

Using a quantitative, theory-driven approach, this paper offers an important expansion of the literature on MAs and low-wage workers. It sheds insight on how healthcare organizations that employ MAs may reconsider their recruitment and retention strategies [12]. As the results show, these strategies should go far beyond simply “paying them more” (given that MAs are historically seen as low-wage workers), because relationships at work and development opportunities are also crucial intervention targets. Additionally, insights on the capabilities as they relate to the four work outcomes highlight the boundaries of previous theorizing on sustainable employability.

## Background

Sustaining the occupational functioning of MAs is challenging given the nature of their work. MAs are typically tasked with routine and administrative tasks [13], and their work can include little variation, challenge, and inspiration. Consequently, MAs may develop concerns about the value of their work to themselves and others [14]. Moreover, given the high demand for MAs, fast-paced work environments, and recent expansions of MA task-profiles, MAs are at risk of being overworked [5]. Their wages, which are comparatively lower than other allied health personnel, have also led to perceptions among MAs as lower status workers within primary care environments [6, 8, 15]. Yet, MAs have core roles to play in care teams and at the work place [16, 17]. Given these considerations, knowledge on how MAs (and other similar low-wage workers) derive value from their work is crucial in sustaining their employability.

### Sustainable employability

Sustainable employability (SE) refers to people’s long-term ability to function at work and in the labor market. Despite varying conceptualizations in the literature, researchers agree that SE covers how well employees can stay in the workforce in the long term. Early research on SE has largely focused on older workers, given the increasing retirement age in European countries [18–22]. Researchers adopted interactionist conceptualizations, positioning SE as an interaction between employees and their environment [9, 10, 23]. Such interactionist conceptualizations, however, have tended to combine cause (i.e., work and aspects of work environment) and effect (i.e., individual employees’ sustainable functioning), therefore complicating the identification of the causal antecedents of SE [24]. Separately, scholars have also positioned SE as an individual characteristic that has multiple dimensions [21, 22, 25]. However, these individual-oriented conceptualizations have tended to underemphasize the role of context and time in SE. The most recent developments in the SE literature therefore state that while SE manifests at the individual level, it inherently *results from* the interaction between the individual and their employment context [26].

Despite the aforementioned limitations, the capability framework [9], which is an interactionist conceptualization, remains widely in use. This framework posits that the SE of workers can be best captured as a set of seven capabilities [9, 10]. Specifically, this framework argues that when workers have a strong set of capabilities they can achieve at work, their employability will be sustained over the long term [9]. The seven capabilities are: (a) using knowledge and skills; (b) developing knowledge and skills; (c) involvement in decision-making; (d) having meaningful relationships at work; (e) setting own goals; (f) earning a good income; and (g) contributing to something valuable. These capabilities also describe the aspects that people typically value in their work that essentially make work worthwhile for them [8].

To address the limitations of the interactionist and individual-oriented conceptualizations, scholars have described that the seven capabilities are better viewed as *predictors*, rather than an outcome, of SE [26]. Specifically, Fleuren and colleagues [26] proposed modeling SE as a composite, individual-level construct that predicts employees’ ability to function at work and on the labor market over time [27]. The authors’ reasoning is that although the seven capabilities have the potential to help employees function in the long term at work, the effect of each capability on employees’ sustained functioning at work varies. By relating the predictors of SE as capabilities, each capability’s effect on SE within a certain work domain or work situation can thus be better identified. In turn, such an approach enables researchers and practitioners to describe the capabilities that are specific to MAs’ ability to function in the context of their employment.

The approach of positioning capabilities as predictors of SE has yet to be widely applied to the health workforce, including those that often referred to as low-wage healthcare workers. Previous studies outside healthcare have considered the relevance of capabilities among gifted employees and people suffering from health conditions like multiple sclerosis [28, 29]. Following the notion that capabilities positively contribute to SE, we hypothesize that capabilities relate positively to SE-indicators and negatively to contra-indicators of SE. Additionally, which capabilities are most relevant to MAs’ SE is of explorative interest in this study, as there is no evidence to date of the relative importance of capabilities among important allied health personnel like MAs.

## Methods

### Design

The present study was based on self-report data from a cross-sectional survey among MAs from a large US-based healthcare organization. The study protocol received ethics approval from the Johns Hopkins University’s institutional review board.

### Procedure

All MAs working in primary care practices and specialty practices affiliated with the participating healthcare organization were invited to participate in the study. Invitations were sent via postal mail to MAs’ workplaces. MAs who opted to participate completed the paper questionnaires and returned them via self-addressed envelopes. MAs who decided not to participate were neither expected to provide any reasons, nor received any follow-up messages. A $5 gift card was included with each invitation as a token of appreciation, regardless of whether the survey was completed.

### Participants

193 MAs were invited to participate and 118 returned the completed questionnaire, resulting in an overall response rate of 61%. The resulting sample of 118 MAs (96% female, 4% male) ranged from 23 to 64 years old (M = 41.58, SD = 11.66). Sixty five percent of participants identified as White, 9% as Black/African American, and 2% as Asian. Fifteen percent of participants identified as Hispanic. These demographics reflect those of the MA population in the US [30]. The average weekly working hours as an MA at their workplace ranged from 8 to 80 h (M = 40.66, SD = 8.75). The number of years that participants have worked in their current occupation ranged from 1 to 42 years (M = 13.72, SD = 10.23), and the number of years they have worked at their current organization ranged from 0 and 42 years (M = 5.09, SD = 6.39). Six participants were excluded for research model estimation due to missing data on relevant variables.

### Measures

The survey included several work characteristics and occupational health variables of MAs, including variables used as indicators for SE (i.e., burnout, job satisfaction, intention to quit, and meaningfulness of work) and the set of seven capabilities.

#### Burnout

Burnout was measured with items from the third edition of the Copenhagen Psychosocial Questionnaire (COPSOQ) [31], which asked the extent to which participants felt “worn out,” “physically exhausted,” and “emotionally exhausted”. An example item was “During the last 4 weeks, how often have you been emotionally exhausted?”. Participants indicated how often they had felt that way during the last four weeks on a scale ranging from one (“not at all”) to five (“all the time”). The mean score across these three items was used as burnout score for each participant. The Cronbach’s alpha for the three-item burnout measure was 0.933, indicating high internal consistency.

#### Job satisfaction

Job satisfaction was measured using a single validated item from the COPSOQ [31]. The specific item formulation was “Regarding your work in general, how pleased are you with your job as a whole, everything taken into consideration?”. Participants indicated their responses on a five-point scale, ranging from one (“very unsatisfied”) to five (“very satisfied”).

#### Intention to quit

Intention to quit was measured using a single validated item from the COPSOQ [31]. Specifically, this item was formulated as “How often do you consider looking for work elsewhere?”. Participants indicated their responses on a five-point scale, ranging from one (“never/hardly ever”) to five (“always”).

#### Meaningfulness of work

Meaningfulness of work was measured using the ten-item Work And Meaning Inventory (WAMI) [32]. The WAMI captures the extent to which participants consider their work meaningful based on their responses to the items on a five-point scale, ranging from one (“absolutely untrue”) to five (“absolutely true”). Example items include “The work I do serves a greater purpose,” “I have found a meaningful career,” and “I have discovered work that has a satisfying purpose.” The Cronbach’s alpha for the WAMI was 0.811, indicating good reliability.

Finally, the seven capabilities were measured using the capability set [10]. This set includes seven capabilities that represent aspects of work employees may value and attain at work. It includes (a) using knowledge and skills; (b) developing knowledge and skills; (c) involvement in decision-making; (d) contributing to something valuable; (e) building meaningful relationships; (f) setting own goals; and (g) earning a good income. For each of these aspects, participants were asked to evaluate: (i) the extent to which they found this aspect of work important; (ii) the extent to which they had the opportunity to attain that aspect; and (iii) the extent to which they actually succeeded at attaining that aspect at work. These three aspects were all scored on scales ranging from one (“not at all”) to five (“very much”). Subsequently, using procedures as detailed in the original paper [10], the presence of each capability would be calculated in such a way that if all three aspects were scored higher than three, the capability would be coded as one (indicating it is present) and zero otherwise (indicating it is absent). For example, for the capability “earning a good income,” participants answered the following questions: (i) “How important is it to you that you can earn a good income in your work?”; (ii) “Does your work offer enough opportunities to do that?”; and (iii) “To what extent do you succeed to actually do that?”.

### Analyses

Before estimating the full model, descriptive statistics and bivariate associations were estimated. Specifically, Phi associations^1^ were estimated between all capabilities, point biserial correlations^2^ were estimated between each capability and each of the SE indicators, and bivariate correlations were estimated between all of the SE indicators using IBM SPSS Statistics 27 [33]. Subsequently, to identify the effects of the capabilities on the indicators of SE in one single model, a structural equation model (SEM) was estimated using Mplus 7 [34]. In this SEM, all four indicators of SE were included as dependent variables. Moreover, each of the capabilities was included as independent variable and linked to each of the dependent variables in the model. As our model did not include any latent variables, our model represented a just identified path model [35].

## Results

### Descriptive statistics

Table 1 provides percentages for the capabilities, Phi associations among the capabilities, and point biserial correlations between capabilities and SE indicators. All capabilities were present among MAs, with the capability of involvement in decision-making being the least common (31.4%), and using knowledge and skills being the most common (64.4%) (Table 1). Additionally, Phi associations suggested that these capabilities co-occurred. The two capabilities of using knowledge and skills and developing knowledge and skills co-occurred most frequently (Phi = 0.636, p < 0.001), whereas involvement in decision-making and earning a good income co-occurred the least frequently (Phi = 0.252, p = 0.006). The association between using knowledge and skills, and earning a good income, was moderate but not significantly different from zero (Phi = 0.340, p = 0.160). Finally, all capabilities had a moderate to small point biserial correlation with the SE indicators (Table 1).

**Table 1:** Overview of Phi associations among the capabilities, percentages of participants with a specific capability per capability, and point biserial correlations between capabilities and sustainable employability indicators

	1	2	3	4	5	6	7
*Capabilities*							
1. Using knowledge and skills	64.40% yes	0.636*** (> 0.001)	0.471*** (> 0.001)	0.350*** (> 0.001)	0.464*** (> 0.001)	0.340 (0.160)	0.420*** (> 0.001)
2. Developing knowledge and skills		59.30% yes	0.517*** (> 0.001)	0.367*** (> 0.001)	0.545*** (> 0.001)	0.420*** (> 0.001)	0.504*** (> 0.001)
3. Involvement in decision-making			31.40% yes	0.445*** (> 0.001)	0.508*** (> 0.001)	0.252** (0.006)	0.487*** (> 0.001)
4. Meaningful relationships				59.30% yes	0.501*** (> 0.001)	0.388*** (> 0.001)	0.480*** (> 0.001)
5. Setting own goals					54.2% yes	0.358*** (> 0.001)	0.516*** (> 0.001)
6. Earning a good income						52.50% yes	0.479*** (> 0.001)
7. Contributing to valuable							55.90% yes
*Sustainable employability indicators*							
8. Burnout	− 0.221** (0.018)	− 0.339*** (> 0.001)	− 0.129 (0.175)	− 0.376*** (> 0.001)	− 0.399*** (> 0.001)	− 0.275** (0.003)	− 0.357*** (> 0.001)
9. Job satisfaction	0.344*** (> 0.001)	0.461*** (> 0.001)	0.358*** (> 0.001)	0.393*** (> 0.001)	0.383*** (> 0.001)	0.499*** (> 0.001)	0.412*** (> 0.001)
10. Intention to quit	− 0.221* (0.017)	− 0.293** (0.001)	− 0.189* (0.042)	− 0.447*** (> 0.001)	− 0.254** (0.006)	− 0.362*** (> 0.001)	− 0.369*** (> 0.001)
11. Meaningful work	0.355*** (> 0.001)	0.486*** (> 0.001)	0.237* (0.011)	0.349** (> 0.001)	0.231* (0.013)	0.290** (0.002)	0.350** (> 0.001)

Numbers above the diagonal represent Phi associations between the dichotomous capability variables and corresponding p-values (bracketed); numbers on the diagonal represent percentages of participants having the specific capability concerned in the sample; numbers in the second part of the table represent point biserial correlations and corresponding p-values (bracketed) between capabilities and the sustainable employability indicators

*p < 0.05; **p < 0.01; ***p < 0.001

Table 2 provides means and standard deviations for the study variables and bivariate correlations among the SE indicators. The standard deviations suggested that considerable variation around the means existed, with intention to quit having the highest magnitude of deviation (SD = 1.15) as compared to meaningfulness of work, which had the lowest magnitude of deviation (SD = 0.63). (Table 2). Additionally, the SE indicators were moderately to highly correlated with each other, with the notable exception of a non-significant relationship between burnout and meaningfulness of work (r = − 0.093, p = 0.327; Table 2).

**Table 2:** Overview of means, standard deviations, and correlations for all sustainable employability indicators

	Burnout	Job satisfaction	Intention to quit	Meaningful work
Burnout	3.35 (1.11)	− 0.525*** (> 0.001)	0.562*** (> .001)	− 0.093 (0.327)
Job satisfaction		3.80 (0.93)	− 0.565*** (> .001)	0.467*** (> 0.001)
Intention to quit			2.26 (1.15)	− 0.338*** (> 0.001)
Meaningful work				4.41 (0.63)

Numbers above the diagonal represent bivariate correlations between the sustainable employability indicators and corresponding p-values (bracketed); numbers on the diagonal represent means and standard deviations (bracketed)

*p < 0.05; **p < 0.01; ***p < 0.001

### Structural equation model (SEM)

**Fig. 1:**
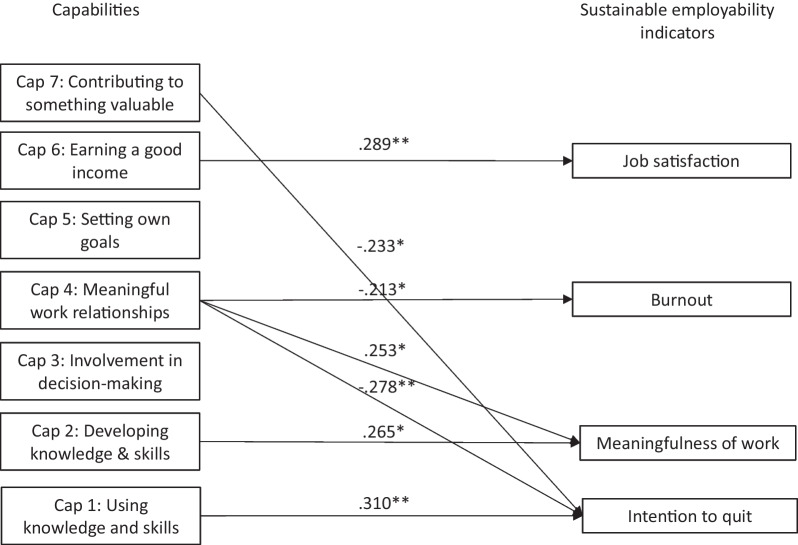
Structural equation model with capabilities predicting sustainable employability indicators and only significant STDYX standardized path coefficients included. *p < 0.05; **p < 0.01; ***p < 0.001)

Table 3 shows all effects estimated in the SEM, which explored the relationships among the capabilities and outcomes of job satisfaction, burnout, intention to quit, and meaningfulness of work. Figure 1 depicts the overall model that includes only the standardized significant effects identified in the SEM. As reported in Table 3 and Fig. 1, the capability of using knowledge and skills related significantly and positively to intention to quit (indicating an undesirable effect, which contrasts with the uncontrolled negative bivariate correlation in Table 1). Moreover, the capability of developing knowledge and skills related significantly and positively to meaningfulness of work. Additionally, having meaningful relationships at work related significantly and positively to meaningfulness of work, and related negatively to burnout and intention to quit (indicating a desirable effect). The capabilities of setting own goals and involvement in decision-making related to none of the dependent variables, and the capability of earning a good income related significantly and positively to job satisfaction only. Finally, the capability of contributing to something valuable related significantly and negatively with intention to quit only. As the SEM represented a just identified path model due to the absence of latent variables, indices indicated a perfect model fit (CFI = 1; TLI = 1; RMSEA = 0; SRMR = 0). Finally, R^2^ values for the dependent variables in the model differed significantly from zero: R^2^_job satisfaction_ = 0.35 (p < 0.001), R^2^_burnout_ = 0.22 (p = 0.001), R^2^_intent to quit_ = 0.29 (p < 0.001), and R^2^_meaningfulness of work_ = 0.24 (p = 0.001).

**Table 3:** Overview of STDXY standardized path-coefficients for each of the paths in the structural equation model including the seven capabilities as independent variables and the sustainable employability indicators as dependent variables

	β	p-value		β	p-value
Cap1 BO	0.043	0.678	Cap1 ITQ	0.310**	0.002
Cap2 BO	− 0.003	0.982	Cap2 ITQ	− 0.088	0.408
Cap3 BO	0.131	0.240	Cap3 ITQ	0.138	0.200
Cap4 BO	− 0.213*	0.044	Cap4 ITQ	− 0.278**	0.005
Cap5 BO	− 0.198	0.060	Cap5 ITQ	− 0.039	0.696
Cap6 BO	− 0.148	0.136	Cap6 ITQ	− 0.152	0.106
Cap7 BO	− 0.158	0.170	Cap7 ITQ	− 0.233*	0.032
Cap1 JS	− 0.066	0.481	Cap1 MW	− 0.031	0.758
Cap2 JS	0.155	0.124	Cap2 MW	0.265*	0.015
Cap3 JS	0.118	0.243	Cap3 MW	− 0.014	0.900
Cap4 JS	0.109	0.257	Cap4 MW	0.253*	0.014
Cap5 JS	0.055	0.572	Cap5 MW	0.007	0.944
Cap6 JS	0.289**	0.001	Cap6 MW	0.059	0.547
Cap7 JS	0.128	0.223	Cap7 MW	0.070	0.540
Correlations specified between the dependent variables					
BO JS	− 0.408***	< 0.001	JS ITQ	− 0.370***	< 0.001
BO ITQ	0.519***	< 0.001	JS MW	0.294**	0.001
BO MW	0.028	0.769	ITQ MW	− 0.142	0.128
Intercepts estimated for the dependent variables					
BO	3.694***	< 0.001	ITQ	2.614***	< 0.001
JS	3.263***	< 0.001	MW	6.753***	< 0.001

All coefficients reported in this table are calculated with STDXY standardization in Mplus 7

Cap1, Capability of using knowledge and skills; Cap2, Capability of developing knowledge and skills; Cap3, Capability of involvement in decision-making; Cap4, Capability of building meaningful relationships; Cap5, Capability of setting own goals; Cap6, Capability of earning a good income; Cap7, Capability of contributing to something valuable; BO, Burnout; JS, Job satisfaction; ITQ, Intention to quit; MW, Meaningfulness of work

*p < 0.05; **p < 0.01; ***significant at p < 0.001

## Discussion

Using a theory-driven approach, this paper empirically tests the relationships between capabilities and indicators of SE among MAs. Using data from US-based MAs, our study suggests that some, but not all, capabilities are relevant to burnout, job satisfaction, intention to quit, and meaningfulness of work. Notably, the capability of building meaningful relationships at work is highly relevant to MAs’ SE, showing desirable relationships with three out of four indicators considered. Earning a good income is positively related to job satisfaction, but not to any of the other indicators considered. Paradoxically, while results from the SEM show that using knowledge and skills is associated with a higher intention to quit, developing knowledge and skills contributes to work being experienced as more meaningful. Finally, contributing to something valuable at work relates negatively to intention to quit. The remainder of this discussion section reflects on each of these main findings, discusses the study limitations, and offers implications for practice.

The most notable finding in this study is the importance of the capability of building meaningful relationships at work for MAs’ SE. With significant desirable effects on three out of four indicators, this aspect of working seems crucial to MAs. It is associated with lower burnout scores, a higher experienced meaningfulness of the work, and a lower intention to quit. This finding aligns with previous studies among healthcare workers that show the importance of the interpersonal and relational aspects of working [36–39]. Additionally, this finding contributes to the literature by showing that meaningful relationships at work are *more* important than other capabilities, including salary, across all indicators except job satisfaction. Our study suggests that investing to help MAs build and maintain relationships—with both coworkers and patients—will be key to enticing them to sustain their employment. Lai and colleagues, for example, have suggested that the routines of conducting patient huddles or rooming can be redesigned to help MAs foster and maintain such relationships at work [40].

The finding that earning a good income is particularly relevant for job satisfaction is in line with existing research on job satisfaction [41], and with studies that examine job satisfaction and pay in particular [42]. Interestingly, earning a good income shows no significant effects for other indicators of SE. This finding suggests that the effect of income on occupational functioning is not universal, but rather depends on the kind of job-related outcomes that researchers target. The SEM approach in the present paper allows us to identify the capabilities—when estimating their effects conjointly—that are most relevant for which indicators. Consequently, this study enables researchers to consider several predictors of and indicators for sustained functioning simultaneously.

A seemingly paradoxical finding based on the SEM is the simultaneous positive effect of “using” knowledge and skills on intention to quit, and the positive effect of “developing” knowledge and skills on meaningfulness of work. This finding is paradoxical as it is often argued that “using” knowledge and skills is the best way of “developing” them [43]. Both capabilities of using and developing knowledge and skills should therefore, in theory, point to desirable outcomes. In this study however, “developing” had positive effects but “using” did not in the SEM. This finding may reflect a situation where one is using their skills and knowledge without being able to develop them, which further speakers to the demands that may be placed on workers (i.e., higher levels of effort are needed to achieve tasks, or the work is more difficult than what the workers typically perform). This explanation also supports why bivariate correlations showed a negative relationship between using knowledge and skills and intention to quit, and why the SEM showed a positive effect of using knowledge and skills on intention to quit, given that all other capabilities were controlled for in the SEM.

Finally, that the capability of contributing to something valuable relates negatively to intention to quit matches the central idea in the capability approach, which posits that work needs to be “valuable” for SE [9]. By showing this relationship between valuable contributions and one’s intention to quit, it seems that MAs who consider their work as having value-add are less likely to want to leave. This notion echoes with existing research on meaningful work [14]. However, it is somewhat surprising that the capability of contributing to something valuable shows no relationship with meaningful work itself in this paper. It is therefore possible that most MAs in this study sample considered their work to be meaningful, but not all of them had the full capability of contributing to something valuable.

## Limitations

A first limitation of this study is its cross-sectional nature. Ideally, studies on SE should employ longitudinal designs and multiple measurements to show the structural effects of work aspects on sustained functioning. Such designs are, however, still rare—particularly in healthcare—due to the complex multidimensional nature of SE, the many facets of work that can be considered, and the general work pressure that healthcare workers face. As such, this limitation does not reduce the value of the present study, but instead calls for a fuller understanding of SE in future research.

A second potential limitation is the reliance on self-report measures. This limitation can be considered problematic because of common method bias [44]. However, modeling a single common method factor [45] in our model resulted in a very poor model fit. This finding, combined with the several mathematical operations that were needed to arrive at the capability scores, suggests that this study is not profoundly affected by common method bias. Additionally, self-report measures are traditionally associated with bias because individuals may have limited self-knowledge, have certain motivations to present themselves favorably, and interpret questions wrongly [46]. However, the constructs used in the present study are largely about what MAs’ individual-level experiences, so much so that limited self-knowledge is not of great concern. Moreover, social desirability bias was limited because researchers outside of their work organizations collected data anonymously, the topics of interest were not generally sensitive, participation was voluntary, and questions could be skipped. Finally, the questionnaire incorporated only validated items and was piloted with MAs to ensure comprehensibility of the questions included. As such, the typical concerns regarding use of self-report measures do not apply very strongly to the present study.

A third limitation of this study is its limited sample that could reduce the generalizability of the findings. Although the sample reflects the demographics of the MA population in the US as a whole, the sample is drawn from a single large health system. Additionally, the findings from this study match previous work that used similar study participants [47, 48] as well as qualitative findings regarding the aspects that MAs value at work [8]. Nonetheless, for a complete picture of the work-related factors that contribute to SE among low-wage workers or health care workers in general, studies incorporating other types of health care personnel are recommended in future research.

## Practice implications

The findings can inform the strategies that healthcare organizations are using to retain MAs, which is important to sustain a cost-efficient and well-functioning primary care clinic. By highlighting the most relevant capabilities (i.e., meaningful relationships at work, contributing to something valuable, earning a good income, and offering opportunities to develop knowledge and skills without demanding heavy skill use), organizations can proactively shape the work environment of MAs, and sustain their employment. In a competitive labor market characterized by shortages [4, 49] and troubled by aftershocks of the “Great Resignation” [50, 51], capitalizing on all the capabilities identified as relevant is recommended. A caveat is that healthcare leaders should not think that simply having a highly social climate at work can compensate for overly low wages. Salaries will still need to be fair to prevent competent MAs from quitting [8].

At the system level, if MAs are considered valuable members of the health workforce, scope of practice regulations will need to be re-examined. Currently, the MA job is rather narrowly defined in the US [52], suggesting overly limited opportunities for development [53, 54]. This study, along with other studies, have identified that MAs want to develop themselves and can contribute more at work [8, 53, 55]. The capabilities can be facilitated by offering MAs more responsibilities and defining the MA role more broadly while staying within scopes-of-practice regulations at the state and organizational level. However, any changes to MAs’ roles will need to match individual MAs’ abilities to avoid creating role stress and other harmful employment conditions [52]. To avoid such mismatch, healthcare organizations will benefit from competency or career development frameworks that clearly state the requirements for skill/career progress, especially as MAs’ can come from various educational backgrounds [56]. Additionally, such development frameworks can foster a common understanding among MAs, other team members, and organizational leaders to facilitate role expansion more effectively. In sum, workplace policies that provide more opportunities for development within the MA-role need to be considered.

## Conclusions

The classification of MAs as low-wage workers may lead health care leaders and managers to use salary increases as the main strategy for talent retention. This study shows that competitive salaries are necessary but not sufficient to motivate MAs to stay and function in their job in the long term. MAs should also have the opportunities to foster meaningful relationships with others, as well as develop competencies at work.

## Data Availability

The datasets generated and/or analyzed during the current study are not publicly available, but are available from the corresponding author on reasonable request.

## Abbreviations

COPSOQ: Copenhagen Psychosocial Questionnaire
MA: Medical assistants
SE: Sustainable employability
SEM: Structural equation model
WAMI: Work and meaning inventory

## Footnotes

1 associations between two dichotomous variables that can be interpreted as bivariate correlations.

2 associations between a dichotomous and a continuous variable that can be interpreted as a mean difference or bivariate correlation.
